# Review of Potential Barriers to Effective Hemostatic Management of Acquired Hemophilia A by Non-Hemophilia Experts in the United States

**DOI:** 10.7759/cureus.33927

**Published:** 2023-01-18

**Authors:** Anjali Sharathkumar, Ali G Mokdad

**Affiliations:** 1 Stead Family Department of Pediatrics, Roy J. and Lucille A. Carver College of Medicine, University of Iowa, Iowa City, USA; 2 Rare Hematology, Takeda Pharmaceuticals U.S.A. Inc, Lexington, USA

**Keywords:** fviii autoantibodies, acquired hemophilia a management, treatment practices, diagnosis, acquired hemophilia a

## Abstract

Acquired hemophilia A (AHA) is an ultra-rare autoimmune disorder caused by autoantibodies against factor VIII. It often presents with life-threatening bleeding to non-hemophilia experts, who have limited awareness of this condition. This review evaluated hemostatic management and identified barriers to optimal management of AHA by non-hemophilia experts in the United States through a literature review. AHA case reports published by non-hemophilia experts from January 2016 through November 2021 in non-hematology journals were critically reviewed for a chronology of clinical course and management, consultation with a hemophilia expert, referencing of available AHA recommendations, discussion of all hemostatic options, and bleed control outcomes; 24 case reports representing 24 patients were identified. Twelve patients had an apparent delay in diagnosis, 17 cases did not seek expert consultation, and 15 did not reference the 2009 International AHA Recommendations, including six in whom hemostatic treatment was not consistent with the recommendations. Of the 17 articles published after the 2017 AHA Guidance, eight did not reference them. Of the five articles published after the 2020 International Recommendations for AHA, three did not reference them. Overall, 14 articles did not discuss all available hemostatic treatment options. Four patients died. Our findings reveal variability in hemostatic management of AHA by non-hemophilia experts in the United States. Lack of AHA awareness remains a primary barrier for optimal management of AHA among non-hemophilia experts. Increasing education about existing AHA guidelines, including available therapies and access to expert care at hemophilia treatment centers, may help improve the outcomes of patients with AHA.

## Introduction and background

Acquired hemophilia A (AHA) is an ultra-rare autoimmune disorder characterized by the development of autoantibodies against coagulation factor VIII (FVIII). These autoantibodies, commonly known as inhibitors, inhibit FVIII activity, impede the activation of the intrinsic coagulation cascade, and impair hemostasis. Patients with AHA typically present with acute bleeding diathesis such as soft tissue hematomas, mucosal bleeding (e.g., epistaxis, gastrointestinal, and urological bleeds), and bleeding within vital organs that can be life-threatening. Bleeding diatheses occur spontaneously or after minor trauma and/or invasive procedures [1-3].

The estimated incidence of AHA is approximately one case per million per year, which may be an underestimation owing to undiagnosed cases [4]. Given the rarity of this condition, a lack of clinical suspicion can delay diagnosis [4], resulting in mortality rates of 9-33% [1,5].

Unlike congenital hemophilia A, which is an X-linked inherited disorder that primarily affects males, AHA is not an inherited disorder that affects both males and females equally [6]. The incidence of AHA increases with age and is more common in elderly patients, with a median age at diagnosis of 73.9 years. In women, another peak occurs during childbearing age (median age, 33.9 years), generally during the peripartum period [6]. About half of AHA cases are associated with other illnesses, such as autoimmune diseases or malignancies, or drugs; the other half is considered idiopathic [4,6]. The pharmacokinetics of AHA inhibitors differ from those of congenital hemophilia A inhibitors. Inhibitors in congenital hemophilia A exhibit type 1 kinetics; they neutralize FVIII at a linear rate correlated with the inhibitor concentration. AHA inhibitors exhibit type 2 non-linear pharmacokinetics; they exhibit a rapid inactivation phase followed by a slower equilibrium phase. During this equilibrium phase, inhibitors are unable to completely inhibit FVIII and residual FVIII activity is often detectable; thus, some hemostatic activity remains, which can lead to misdiagnosis and treatment [1,7]. Gaining a deeper understanding of the type 2 pharmacokinetics of inhibitors in AHA is critical for clinical management [8].

The treatment of AHA relies on two main principles: (i) control of acute bleeding diathesis and (ii) eradication of the inhibitor [9]. Acute bleeding diathesis can be treated with hemostatic agents, including factor replacement with recombinant porcine FVIII (rpFVIII), bypassing agents such as recombinant factor VIIa (rFVIIa) or activated prothrombin complex concentrate (aPCC) (off-label in the United States), and other agents such as human FVIII or desmopressin [8]. Eradication of the inhibitor requires immunosuppressive therapies such as steroids, cyclophosphamide, and/or rituximab [10].

To help clinicians manage these patients, several expert recommendations for the diagnosis and treatment of AHA have been published [8,9,11]. However, most patients with AHA typically present to physicians who do not have experience in managing patients with hemophilia (e.g., emergency room physicians, geriatricians, obstetricians, oncologists, rheumatologists, surgeons) [5], and their level of awareness of these recommendations is unknown. Therefore, we sought to investigate potential barriers to and suggest solutions for the optimal management of AHA by non-hemophilia experts in the United States via a literature review.

We critically reviewed case reports of AHA published by non-hemophilia experts in non-hematology journals over the last five years. Specifically, we focused on assessing patient outcomes, with particular emphasis on the initial control of bleeding (e.g., chronology and type of hemostatic agents used, when this information was available). We further investigated whether these treaters consulted a hemophilia expert and referenced and/or followed the published recommendations for AHA.

**Figure 1:**
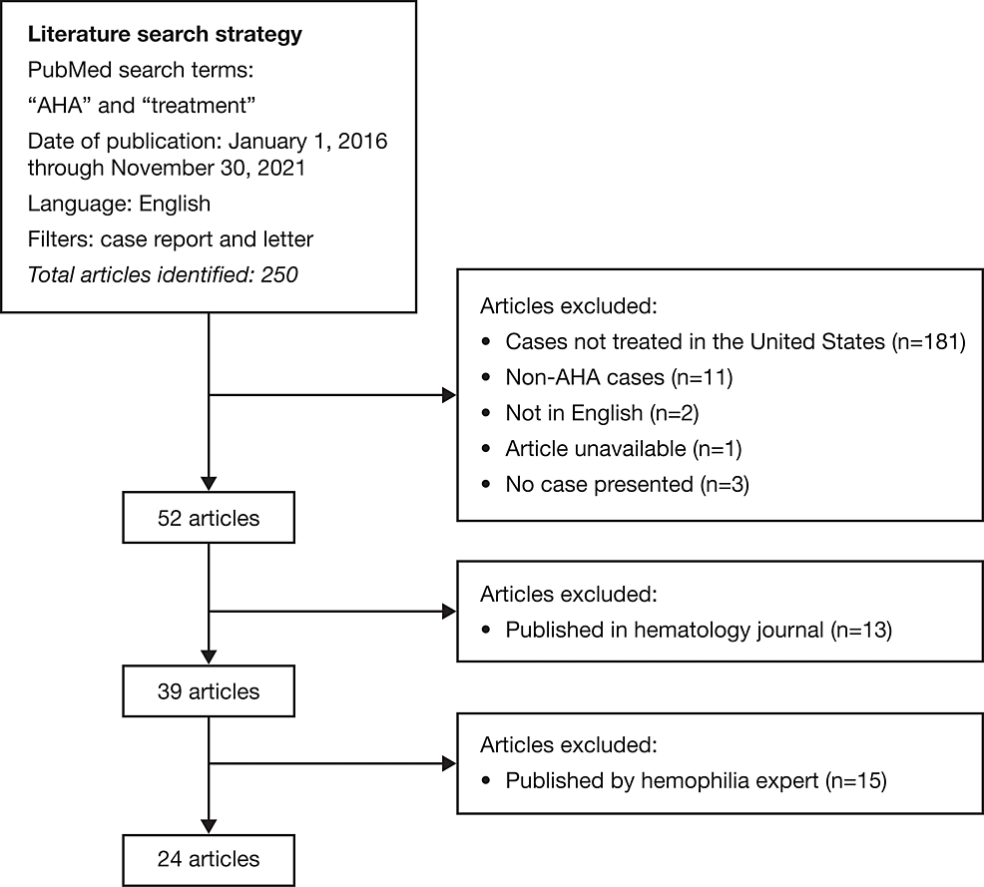
Literature search strategy flow chart AHA, acquired hemophilia A.

A literature search was conducted of articles published in the PubMed electronic database (Figure 1).

The search strategy included the terms “acquired hemophilia A” and “treatment,” and was restricted to articles in English published from January 2016 through November 2021. This range was chosen to reflect the period during which all currently recommended first-line hemostatic options for AHA treatment were available in the United States [8].

AHA case reports and letters have been included. The exclusion criteria were as follows: (i) patients treated outside the United States, (ii) articles not pertaining to AHA, (3) articles not written in the English language, (iv) articles not describing a clinical case, (v) unviable articles, (vi) articles published in hematology journals (to focus on articles targeting a non-hemophilia-expert audience), and (vii) article with authors who appeared to be hemophilia experts (defined as a hematologist with experience managing patients with hemophilia either at a hemophilia treatment center (HTC) or at a hematology clinic), based on author affiliations, internet research, and review of the author’s disease expertise). Examples of non-hemophilia experts include emergency room physicians, obstetricians, gynecologists, and surgeons. Hematology/oncology-affiliated authors were included, as they were not necessarily considered hemophilia experts unless there was evidence of bleeding disorder expertise, as evaluated by manual author review. Only articles published in the United States were included because of the potential differences in healthcare delivery systems, including access to HTCs. Moreover, the availability of all treatment options may not have been the same worldwide relative to the start date of our literature search.

The following data were extracted: patient demographics, clinical presentation, medical history, hemostatic regimens used, and author specialties (based on affiliations and internet research). To assess AHA management awareness, the following areas were evaluated: (i) any apparent delay in diagnosis, which was assessed subjectively based on treatment chronology and interventions (e.g., if the patient received another diagnosis before AHA, if the patient was treated with non-hemostatic treatments until AHA was discovered), (ii) chronology of clinical course and type of treatment intervention/s used to control acute bleeding, (iii) consultation with a hemophilia expert, (iv) referencing of available recommendations and guidance for AHA diagnosis and treatment based on the date of publication, specifically those published by Huth-Kühne et al. in 2009 [11], Kruse-Jarres et al. in 2017 [8], and Tiede et al. in 2020 [9], (v) whether the initial hemostatic treatment was consistent with the available recommendations for hemostatic control, (vi) whether all available acute hemostatic treatment options were discussed in the report, and (vii) control of bleeding diathesis and mortality outcomes.

## Review

Of the 250 articles initially identified in our literature search, 198 were immediately excluded based on the criteria described earlier (Figure 1). Thirteen of the 52 reports were published in hematology journals and 15 of the remaining 39 were published by hemophilia experts. A total of 24 articles fulfilled the inclusion criteria (data summarized in Table 1).

**Table 1:** Summary of 24 acquired hemophilia A case reports published in non-hematology journals by non-hemophilia expert authors * Some articles had authors of more than one specialty. † Some patients received more than one blood support product or hemostatic agent. ‡ Eight of 17 articles that were published in 2018 and onward after the publication of the Kruse-Jarres et al., 2017 AHA Guidance [8]. § Three of five articles that were published in 2021 after the publication of the Tiede et al., 2020 AHA International Recommendations [9]. 4F-PCC: 4-factor prothrombin complex concentrate; AHA: acquired hemophilia A; aPCC: activated prothrombin complex concentrate; FFP: fresh frozen plasma FVIII: factor VIII; rFVIII: recombinant factor VIII; rFVIIa: recombinant factor VIIa; rpFVIII: recombinant porcine FVIII

Patient characteristics	
Number of patients in 24 articles	24
Median age (years)	68 (range: 21–91)
Male to female ratio	5:3
Author specialties (n=98)^*^	
Internal medicine	38
Hematology/oncology	29
Pathology	8
Oral and maxillofacial surgery	4
Cardiology	3
Intensive care trauma surgery	3
Pharmacy	3
General practitioner/family physician	3
Gastroenterology	1
Hospice and palliative care	1
Radiology	1
Pulmonary and critical care medicine	1
Clinical oncology	1
Rheumatology	1
Anesthesiology	1
Endocrinology	1
Infectious diseases	1
Allergy and immunology	1
Hemostatic agents used (number of patients)^†^	
Blood product support [packed cell transfusion, platelets, FFP, cryoprecipitate]	19
rFVIIa	13
aPCC	12
rpFVIII	4
FVIII	3
rFVIII	2
Emicizumab	2
4F-PCC	1
Outcomes (number of patients; n=24)	
Bleeding controlled	20
Bleeding controlled with multiple agents	11
Bleeding controlled with multiple agents and significant delay	7
Controlled with significant delay	9
Bleeding uncontrolled (death)	4
Potential issues with diagnosis or treatment (number of articles)	
Apparent delay in diagnosis	12
All treatment options not discussed	12
Did not reference 2009 International AHA Recommendations [11]	15
Did not reference 2017 AHA Guidance [8]	8^‡^
Did not reference 2020 International Recommendations for AHA [9]	3^§^
Did not reference any guidelines	7
Did not appear to follow recommendations on bleed control	11
Did not seek hemophilia expert consultation	17

Of the 98 authors, 18 specialties were represented, with internal medicine being the most common (Table 1). The patient characteristics and management history are detailed in the Appendices.

**Figure 2:**
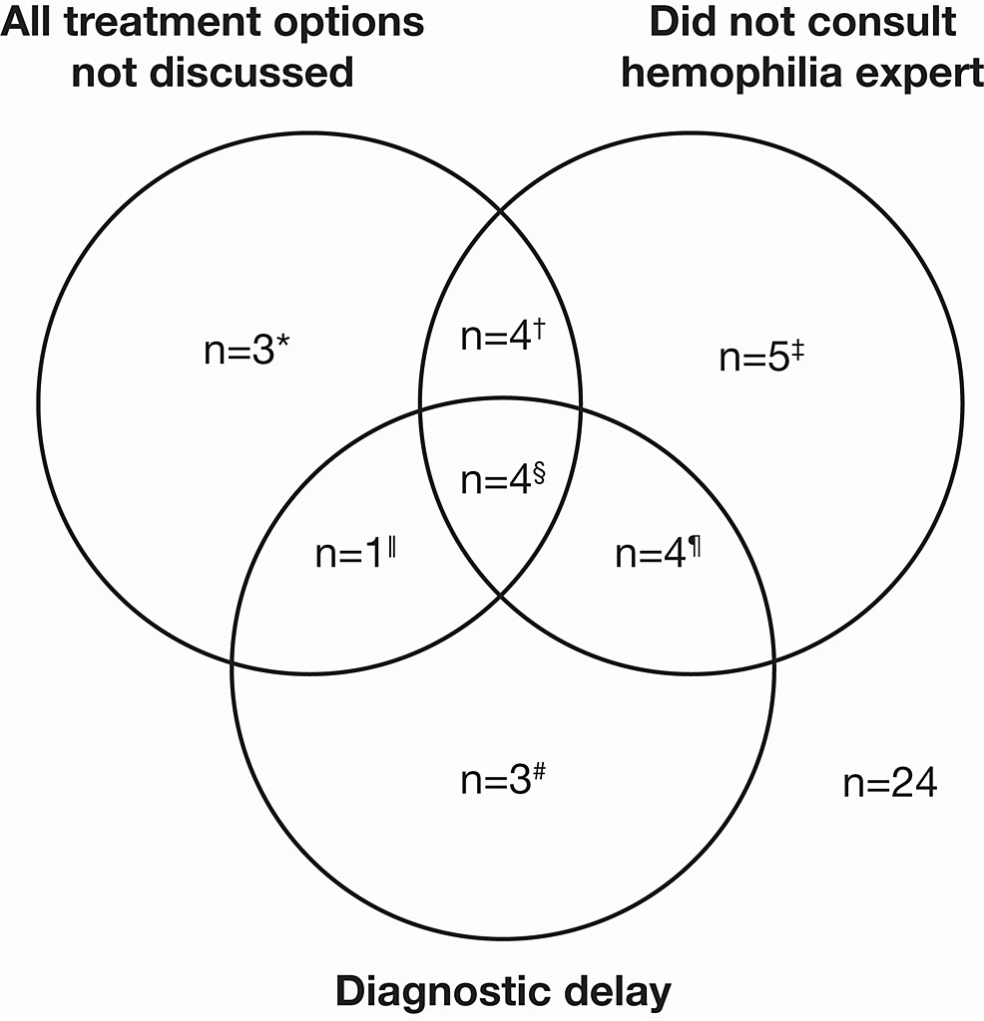
Overlap between a lack of hemophilia-expert consultation, discussion of treatment options, and apparent delay in diagnosis in 24 AHA case reports published by non-hemophilia-expert authors. *Three studies [16,17,26]. †Four studies [13,14,18,23]. ‡Five studies [21,27-30]. §Four studies [12,15,19,22]. ǁOne study [31]. ¶Four studies [24,25,32,33]. #Three studies [20,34,35]. n: the number of studies; AHA: acquired hemophilia A

Seventeen of the 24 articles did not mention consultation with a hemophilia expert (Table 1). Fifteen of the 24 studies did not reference the 2009 international AHA recommendations (Table 1) [11]. Twelve of the 24 articles did not discuss all recommended first-line hemostatic treatment options (Table 1), and seven of these 12 did not cite the 2009 International AHA recommendations [12-18]. In the subset of articles that were published after the publication of the 2017 AHA guidance [8], eight out of 17 did not cite them [14,16,19-24], and five of eight also did not discuss all available recommended first-line hemostatic treatment options [14,16,19,22,23]. Three out of five articles published in 2021 that could have referenced the 2020 international AHA recommendations [9] did not [18,24,25]. Twelve cases appeared to be associated with a delayed diagnosis (Figure 2).

Nine of these 12 patients either did not discuss all treatment options or did not mention consultation with a hemophilia expert (or both). Bleeding appeared to be controlled in 20 cases, although multiple hemostatic agents were required in 11 of 20 cases [12,16,18,21,23-25,27-30,34] and considerable time was required to achieve bleeding control (hospitalization of ≥7 days) in nine of 20 cases [16,18,21,23-26,29,34].

Four of the 24 patients (17%) in our review died: one because of suspected intracranial hemorrhage (without autopsy confirmation, so a thrombotic event was not excluded; treated with aPCC and rFVIIa) [22]; one because of pulmonary embolism possibly attributed to aPCC treatment (dose unspecified) [15]; and one because of cardiac arrest following a stroke (treated with FVIII, rFVIIa, and rpFVIII; Table 1) [20]. The fourth patient died from progressive shock with renal and hepatic failure following cardiac arrest 23 days after admission; bleeding remained uncontrolled despite treatment with 4-factor prothrombin complex concentrate, recombinant FVIII (rFVIII), and rFVIIa, and a pulmonary embolism eventually developed after two days of rFVIIa therapy (30 µg/kg), prompting a switch to plasma exchange [32]. Based on our assessment, all 24 cases either did not discuss all treatment options, did not consult a hemophilia expert, and/or had a diagnostic delay, and all four patients who died had a delay in diagnosis (Figure 2). Details of case reports published by non-hematology experts that did not discuss all available first-line hemostatic options are available in the Appendices.

Discussion

Our study shows that there is variability in the hemostatic management of AHA among non-hemophilia experts. Our analyses revealed apparent delays in diagnosis in several cases, consistent with the findings of the European Acquired Haemophilia Registry [6]; this registry captured data from 117 centers in 13 European countries and reported a median delay in AHA diagnosis of three days (interquartile range, 0-12 days) after the patient presented with bleeding diathesis. Although these delays might appear relatively short, they could still contribute to poor clinical outcomes, given that most patients presenting with AHA are elderly, have significant comorbidities, and may have life-threatening bleeds [6]. Notably, in our review, one of the patients who died was undiagnosed for two years and had previously presented with life-threatening acute gastrointestinal bleeding [22].

While the identified variability in hemostatic management may have resulted from a lack of awareness of AHA itself, we also noted that most of these clinicians may not be cognizant of HTCs as an important resource for the management of patients with bleeding disorders. HTCs are specialized multidisciplinary healthcare centers that use a team-based shared decision-making process to improve patient outcomes and offer integrated and comprehensive diagnostic and treatment services (including counseling, case management, care coordination, outreach, research, surveillance, and outpatient pharmacy services) to patients with hemophilia and other bleeding disorders [36].

Based on our assessment of potential barriers to the optimal care of people with AHA by non-hemophilia experts, we outlined potential solutions or resources to help physicians in these settings (Table 2).

**Table 2:** Potential resources to help overcome the barriers to effective management of acquired hemophilia A AHA: acquired hemophilia A; anti-pFVIII: anti-porcine FVIII; aPTT: activated partial thromboplastin time; CDC: Centers for Disease Control; ELISA: enzyme-linked immunosorbent assay; FVIII: factor VIII; HTC: hemophilia treatment center; rFVIIa: recombinant activated factor VIIa; rpFVIII: recombinant porcine factor VIII

Potential barrier	Potential solution or resources to overcome barrier
Diagnosis	
Lack of familiarity with AHA symptoms and differential diagnosis; isolated aPTT with normal prothrombin time should prompt differential diagnosis. A hemophilia expert might recommend a mixing assay and an assay to confirm the presence of inhibitors (Bethesda assay, Nijmegen-Bethesda assay, or an anti-FVIII antibody ELISA)	Consult with local hematologist/hemophilia expert (CDC Directory for HTCs) AHA recommendations (https://onlinelibrary.wiley.com/doi/full/10.1002/ajh.24777) [8]
Access to fast and reliable laboratory assays like the mixing assay, and assays to confirm the presence of inhibitors (Bethesda assay, Nijmegen-Bethesda assay, or an anti–FVIII antibody ELISA)	Consult with local hemophilia expert
Access to appropriate hemostatic medications	
Lack of familiarity with treatment options and considerations when choosing a hemostatic agent	Consult with local hematologist/hemophilia expert and the AHA consensus recommendations (https://onlinelibrary.wiley.com/doi/full/10.1002/ajh.24777) [8]
Lack of availability of recommended first-line hemostatic agents rpFVIII and bypassing agents	In many institutions rpFVIII can be obtained in 4 to 6 hours by a delivery service (contact: BioCARE at 1-800-304-3064) and can be available at some HTCs rFVIIa can be obtained by contacting a 24/7 service (tel: +1-877-NOVO-777)
Access and reimbursement education	Manufacturers have patient support teams that can provide healthcare providers with access and reimbursement education to help facilitate patient access to prescribed treatments https://www.novosevenrtpro.com/ Takeda Hematology Support Center | For Healthcare Professionals (hematologysupportpro.com)
Misconception that anti-pFVIII inhibitor assays are required before initiating rpFVIII treatment	The rpFVIII prescribing information states to “consider evaluating for presence of anti-rpFVIII antibodies prior to initiation of treatment” [37]. The activity of rpFVIII can be assessed by standard FVIII assays and by observing clinical response [10,38]. An algorithmic approach to dosing rpFVIII developed by Ellsworth et al., can be used which measures FVIII recovery alone to predict rpFVIII treatment efficacy early on, guide dosing and regimen and in turn, increase safety [38].

The most important step for non-hemophilia experts to take if they encounter a patient with bleeding symptoms is to immediately screen with standard coagulation tests, if readily available (including prothrombin time and activated partial thromboplastin time (aPTT), and assessment of FVIII and factor IX levels) [8]. Proper interpretation of coagulation laboratory test results is critical; however, physicians do not always recognize the potential clinical significance of abnormal values, specifically the isolated prolongation of aPTT in patients with AHA. Reding et al. conducted a survey of 302 physicians across several specialties, including internal medicine, emergency medicine, rheumatology, hematology-oncology (17% of the sample), and critical care medicine, to identify potential barriers to the effective recognition and management of AHA [37]. The authors concluded that a large proportion of physicians did not recognize prolongation of aPTT as an indicator of underlying coagulopathy in a patient presenting with bleeding; hence, they were unlikely to investigate the cause of bleeding. Moreover, over half of the physicians would not have chosen to repeat aPTT testing to confirm the initial result. Notably, neither emergency medicine nor critical care physicians considered coagulopathy as the primary explanation for the clinical presentation of the patient in question, and physicians were also reluctant to consult a hemophilia expert. This survey and our own findings suggest that more emphasis should be placed on collaboration between non-hemophilia and hemophilia experts, which may help educate providers about the type of coagulation tests to order and their correct interpretation.

In many of the identified articles, physicians might have been unaware of all available hemostatic treatment options (especially the availability of rpFVIII) or unclear of how they should be dosed [15]. In 2017, Kruse-Jarres et al. provided comprehensive guidance for the management of AHA, including recommended first-line hemostatic treatments and dosing regimens [8]. Briefly, these therapies can be divided into two major categories: (i) FVIII-based therapy (rpFVIII), and (ii) bypassing therapies (aPCC and rFVIIa) [38,39].

rpFVIII is a recombinant analog of porcine FVIII and has enough homology to human FVIII to effectively substitute for endogenous FVIII in the coagulation cascade while remaining unrecognized by FVIII inhibitors because of differences in protein epitopes. rpFVIII has a recommended initial dose of 200 U/kg [40], which is thereafter adjusted in terms of dose and frequency based on FVIII recovery levels and individual clinical response (although real-world experience indicates that an initial dose of 100 U/kg is sufficient) [41]. An algorithmic approach to dosing with rpFVIII, depending on FVIII recovery levels, can be used to guide dosing and does not require rpFVIII inhibitor assay results prior to treatment initiation [41]. Although continued treatment with rpFVIII might cause an anamnestic reaction due to inhibitor cross-reactivity, rpFVIII remains effective in the majority of patients with AHA with pre-existing or de novo inhibitors [40,42]. Most importantly, the activity of rpFVIII can be quantified using the one-stage FVIII clotting assay, which can guide dose titration or therapy switching [40].

Bypassing agents achieve hemostasis by generating thrombin (in the absence of FVIII) at the site of bleeding [10] and are recommended for non-life-threatening or non-limb-threatening bleeding [8]. rFVIIa is a recombinant form of human FVIIa with a recommended dosing frequency of 70-90 µ/kg every two to three hours until hemostasis is achieved. aPCC is a plasma-derived concentrate that contains zymogen forms of procoagulant factors (F) II, FVII, FIX, and FX with trace amounts of their activated forms and anticoagulant protein C in a physiologically balanced ratio (off-label in the United States) [43]. Unlike rpFVIII, the hemostatic activity of both aPCC and rFVIIa cannot be measured by standardized and widely available assays; therefore, it is not possible to predict treatment response or adjust the dose accordingly [8].

The 2017 AHA Guidance recommended rpFVIII as an appropriate first-line replacement therapy for life- or limb-threatening bleeding, where the drug is readily available if no baseline rpFVIII inhibitor is present and if FVIII activity measurement is readily available [8]. Unlike bypassing therapies, rpFVIII may not be readily available to all providers. However, manufacturers often provide options to facilitate swift access to products including rpFVIII (Table 2). The 2017 AHA guidance recommends bypassing aPCC or rFVIIa therapies for non-life-threatening or non-limb-threatening bleeding [8]. Half of the treaters identified in this review used bypassing agents, whereas only four providers used rpFVIII. Although bypassing agents have an established history of efficacy and safety for the treatment of AHA, rpFVIII effectively controls bleeding. It has the additional advantage of a longer half-life compared with rFVIIa [40] and may have a lower risk of thrombotic complications compared with bypassing agents, as its activity can be measured and doses can be adjusted accordingly during treatment. One of the patients in our review endured a 21-day complicated hospital stay, during which a >500% peak in FVIII levels was reached during rFVIII treatment (despite dose de-escalation), which presented a high risk of thrombosis [34]. The ability to measure rpFVIII levels throughout treatment, not just when the inhibitor was eradicated, may have been beneficial in this case.

Few providers used rpFVIII. Although we were unable to clearly identify barriers to the utilization of rpFVIII, it is possible that physicians were aware of this treatment option but were concerned about the potential for the development of anti-porcine FVIII (anti-pFVIII) antibodies, as acknowledged in the prescribing information and in the 2017 AHA guidance [8,40]. The rpFVIII pivotal phase 2/3 study allowed for the inclusion of patients with anti-pFVIII inhibitor ≤20 BU and 10 such patients were included, with 9/10 and 1/10 achieving an effective or partially effective response at 24 hours, respectively. One of these patients who achieved an effective response at 24 hours was later discovered to have had a baseline anti-pFVIII titer of 29 BU. In addition, five patients developed de novo anti-pFVIII inhibitors in this study, and all of them had effective responses to rpFVIII at 24 hours [42]. Another study has further demonstrated continued hemostatic control with rpFVIII even in the presence of anti-pFVIII inhibitors, which suggests a possible lack of direct correlation between rpFVIII inhibitor presence and treatment efficacy [41]. This may reflect the type 2 kinetics of the acquired inhibitor, which may allow rpFVIII to remain clinically effective despite the presence of anti-pFVIII inhibitors [1,7]. Another barrier to rpFVIII treatment might be the misconception that anti-pFVIII antibody titers must be obtained prior to rpFVIII treatment [40]. Although treaters are encouraged to consider evaluating anti-pFVIII inhibitor titers before using rpFVIII [40], results from these assays are not required before treatment initiation, as FVIII activity can be measured using the standard FVIII activity one-stage clotting assay (OSCA) and the dose adjusted accordingly (Table 2) [10,41].

Four patients (17%) identified in our literature review died [15,20,22,32]. Although AHA is acknowledged to have a high mortality rate [8], there may be indications of some lack of knowledge and awareness about AHA and its optimal therapies that may have negatively affected the clinical outcome. Clearly, there were delays in diagnosis and three [15,20,22] of four reports did not mention the dosing regimens used for bypassing agents. For example, Roy et al. did not provide doses of both bypassing therapies used or specified if they were administered sequentially or concomitantly [22]; these agents have a known potential for thrombotic adverse events [38,39]. It is particularly important to take caution with the dosing of bypassing agents, as their activity cannot be monitored with readily available assays [8]. Indeed, experts have acknowledged that concern about thrombosis risk associated with bypassing agents may have led to the underdosing of these agents [38,39,44]. This patient was also treated intermittently for two years for gastrointestinal bleeding and had inconclusive endoscopic studies before diagnosis [22], clearly demonstrating the issue of delayed diagnosis of AHA in the United States. Aslam et al. described a patient with AHA who died of pulmonary embolism associated with aPCC treatment [15]. However, the authors did not provide the dose or frequency of aPCC dosing. Moreover, the authors did not appear to seek hemophilia-expert input and did not mention rpFVIII as a treatment option, despite having referenced the 2017 AHA guidance.

Two elderly patients (91 and 87 years old) with a high thrombotic risk (a history of hypertension, atrial fibrillation, mitral stenosis) were treated with emicizumab after achieving hemostatic control with bypassing agents rFVIIa and aPCC, both of which increase the risk of thrombosis [12,17]. rpFVIII might be a better treatment choice for these types of patients because it can be monitored with the OSCA before treatment with emicizumab or with the bovine chromogenic assay after treatment with emicizumab [45]. Moreover, emicizumab is not currently approved for AHA treatment in the United States, and could potentially cause thrombotic complications, especially in elderly patients who have underlying comorbid conditions [46].

Since the beginning of the coronavirus disease 2019 (COVID-19) outbreak, a few cases of severe acute respiratory syndrome coronavirus 2 (SARS-CoV-2) infection associated with de novo AHA have been reported in the literature [47-50]. COVID-19-infected patients have been shown to develop significantly more thrombotic complications and to have increased von Willebrand factor (VWF) activity, VWF antigen, and FVIII activity levels, as well as elevated D-dimer and fibrinogen levels, compared with non-COVID-19-infected patients [51]. In one case we reviewed, the physician administered rFVIIa followed by aPCC in a patient with COVID-19 despite the heightened thrombotic risk due to COVID-19 infection [50]. Although one of the authors of this case report is a hemophilia treater and we did not include it in our overall analysis, moving forward, we feel it is important and relevant to carefully consider the appropriate management of AHA in patients with SARS-CoV-2 or similar thrombogenic infections that increase thrombotic risk.

Our critical review has some limitations. We confined our review to the case details reported in previously published papers. Therefore, it is possible that some of our assumptions regarding potential knowledge gaps or choices of treatment and consultation with hemophilia experts may not be accurate. Furthermore, we made assumptions about the availability of treatment options and published recommendations based on the publication date of the articles, which are subject to some uncertainty. We also assumed that articles that did not discuss other treatment options did not consider them at all. While this might not be accurate in every case, the articles in which bleeding was controlled with multiple agents [12,17,18,21,23-25,27-29,34] might have achieved better outcomes sooner with a more appropriate choice of first-line agent and, in the cases in which the patient died, not all available recommended agents were tried [15,22,32]. Moreover, we did not compare our findings with a similar analysis of publications by hemophilia experts.

This review lends weight to previous calls to raise the level of awareness of AHA among non-hemophilia experts practicing in the United States and to encourage them to consult with hemophilia experts to assist with diagnosis and treatment [5]. The importance of expert consultation regarding the management of patients with bleeding disorders is recognized by the Medical and Scientific Advisory Council of the National Hemophilia Foundation guideline documents 257 [52] and 265 [53]. These guidelines recommend an ongoing relationship with a bleeding disorder specialist during the perinatal care of women and consultation with a bleeding disorder specialist for the care of patients presenting at the emergency department with bleeding disorders. Moreover, the 2017 AHA guidance states that patients with AHA are best managed or treated in close consultation with physicians experienced in AHA [8].

## Conclusions

Our findings indicate variability in hemostatic management by non-hemophilia experts in the United States. They also revealed potential barriers to AHA diagnosis and treatment; we have suggested resources that may help overcome these barriers. Non-hemophilia experts are urged to consult with local hemophilia experts with experience treating AHA in the diagnosis and treatment of patients with suspected or confirmed AHA and to follow the most recent AHA guidelines. Finally, it is apparent that more efforts to help raise the level of awareness of AHA among non-hemophilia experts in the United States are urgently needed to improve clinical outcomes for these patients.

## Appendices

**Table 3:** Details of case reports published by non-hematology experts that did not discuss all available first-line hemostatic options. * Time to bleed control was not reported in any articles. † Blood support product used. ‡ Multiple agents defined as use of ≥2 hemostatic agents. § Significant clinical delay defined as hospitalization of ≥7 days. AE: adverse event; AHA: acquired hemophilia A; aPCC: activated prothrombin complex concentrate; aPTT: activated partial thromboplastin time; BU: Bethesda units; F: female; FVIII: factor VIII; FFP: fresh frozen plasma; ICH: intracerebral hemorrhage; IM: intramuscular; IV: intravenous; M: male; N/A: not applicable; PRBC: packed red blood cells; Q#H: every # hours; QD: daily; RBC: red blood cells; rFVIIa: recombinant activated factor VIIa; rpFVIII: recombinant porcine factor VIII; SC: subcutaneous.

Citation	Patient age (years)	Patient sex	Bleeding location	FVIII activity and inhibitor levels at diagnosis	Hemostatic regimens used	Outcomes	
						Bleeding control^*^	Overall
Al-Banaa et al., 2019 [12]	87	F	Pelvic, breast, and chest wall hematomas	FVIII activity <1 FVIII inhibitor >100 BU	aPCC Emicizumab	Controlled with multiple agents^‡^	Follow-up for two months after hospitalization while continuing emicizumab treatment No reported AEs or complaints of major bleeding
Williams et al., 2017 [13]	75	M	Left arm	FVIII activity 0% FVIII inhibitor 18.4 BU	PRBC^†^ rFVIIa	Controlled	Durable remission over three years
Gokozan et al., 2019 [14]	76	M	Extensive bruising on left side of body and hematuria	FVIII activity <1% FVIII inhibitor 31 BU	rpFVIII 100 U/kg	Controlled	At discharge, managed with aPCC 100 U/kg daily until inhibitor resolved
Aslam et al., 2020 [15]	64	F	Left thigh	FVIII activity <6% FVIII inhibitor N/A	PRBC^†^ 2 U FFP QD 80 U/kg FVIII concentrate QD aPCC	Uncontrolled (death)	Patient died of a suspected pulmonary embolism
Pinchover et al., 2020 [16]	77	M	Soft-tissue hematoma along right posterior chest wall	FVIII activity: “low”; value not reported FVIII inhibitor: Bethesda titer positive; value not reported	PRBC^†^ (5 U)	Controlled with significant clinical delay	Continued daratumumab and dexamethasone with FVIII and aPTT improvement to 33 IU/dL and 119 s, respectively, within four weeks after discharge
Hess et al., 2020 [17]	91	M	Large IM hematoma in iliopsoas muscle secondary to continued bleeding	FVIII activity <1% FVIII inhibitor 44 BU	rFVIIa Emicizumab	Controlled	Bleeding was controlled ultimately with emicizumab and remained controlled when treatment stopped
Bin Waqar et al., 2021 [18]	31	F	Injection-site hematoma over the triceps and the site of the incision after delivery of baby via cesarean section Profuse bleeding from IV lines Hematoma anterior to the uterus’s surgical line and 2 other hematomas within the SC soft tissues with IM hematoma	FVIII activity <2% FVIII inhibitor 72 BU	FFP aPCC rFVIIa RBC transfusions	Controlled with multiple agents and significant clinical delay	Discharged home on aminocaproic acid 10 days after delivery
Kaur et al., 2018 [19]	78	F	Mouth, tongue, chest, upper extremities	FVIII activity <1% FVIII inhibitor 59.7 BU	PRBC 1 U^†^	Controlled	No further bleeding episodes; remained asymptomatic at six months
Roy et al., 2020 [22]	74	F	Melena and left pectoral hematoma	FVIII activity <1% FVIII inhibitor 91 BU	aPCC rFVIIa	Uncontrolled (death)	Died from suspected ICH (autopsy was not performed, and a thrombotic event was not excluded)
Chung et al., 2021 [23]	65	M	Bilateral, upper-limb, soft-tissue hematomas requiring urgent fasciotomy on right arm for compartment syndrome Bleeding from IV sites and continuous oozing from surgical wound	FVIII activity undetectable FVIII inhibitor 176 BU	PRBC^†^ Cryoprecipitate platelet transfusions FFP FVIII products rFVIIa	Controlled with multiple agents and significant clinical delay	Hospital day 28 FVIII levels normalized, and inhibitor was undetectable three months after discharge, patient presented to outpatient hematology clinic and had normal FVIII level and aPTT with complete resolution of AHA
Rivera Cora et al., 2017 [50]	90	F	Left arm toward thorax, left thigh	FVIII activity <11% FVIII inhibitor 76 BU	PRBC^†^ FFP Cryoprecipitate rpFVIII 12,000 U IV Q6H ×4 doses	Controlled	Discharged after 29 days with home care planning

**Table 4:** Patient medical and treatment history 4F-PCC: 4-factor prothrombin complex concentrate; AD: admission day; AE: adverse event; AHA: acquired hemophilia A; aPCC: activated prothrombin complex concentrate; aPTT: activated partial thromboplastin time; BSA: body surface area; BU: Bethesda units; CKD: chronic kidney disease; COPD: chronic obstructive pulmonary disease; CT: computed tomography; DNR: Do Not Resuscitate; ED: emergency department; F: female; FFP: fresh frozen plasma; FVIII: factor VIII; GI: gastrointestinal; Hb: hemoglobin; ICH: intracranial hemorrhage; IM: intramuscular; IV: intravenous; IVIg: intravenous immunoglobulin; M: male; MRI: magnetic resonance imaging; PCC: prothrombin complex concentrate; PO: per os (orally); PRBC: packed red blood cells; PT: prothrombin time; PTT: partial thromboplastin time; Q#H: every # hours; Q#W: every # weeks; rFVIIa: activated recombinant factor VII; rFVIII: recombinant factor VIII; rpVIII: recombinant porcine FVIII; SC: subcutaneous; T2DM: type 2 diabetes mellitus; TPEx: therapeutic plasma exchange; U: units. *Dosing regimen not provided.

Citation	Patient age, years/sex	Medical history (comorbid conditions)	Clinical presentation and site of bleeding diathesis	Hemostatic regimens for control of acute bleeding diathesis	Maintenance hemostatic therapy		Treatment for inhibitor eradication	Clinical course and outcomes during post-discharge follow-up
Al-Banaa et al., 2019 [1]	87/F	Chronic atrial fibrillation, symptomatic anemia	Hematomas at multiple anatomical sites: pelvic region; left chest wall extending to left upper extremity, axillary and breast area	aPCC 50 U/kg Q12H for two weeks emicizumab 1.5 mg/kg/week	Yes: aPCC discontinued, patient was discharged, and bypass treatment was stopped Transitioned to emicizumab 3.0 mg/kg/week for 1 month, then 1.5 mg/kg/week		Not given	Total follow-up: two months after hospitalization while continuing emicizumab treatment No reported AEs or major bleeding diathesis reported
Al-Shbool et al., 2018 [2]	71/F	Hypertension, abdominal and thoracic aortic aneurysm, COPD	Large SC hematoma with IM bleeding into anterior compartment of right thigh	rFVIIa 5 mg multiple times daily rpFVIII 15,000 U twice daily	No clinical improvement after treatment with rFVIIa and rpFVIII (and methylprednisolone 80 mg/day)		Cyclophosphamide chemotherapy and rituximab initiated^*^ After 2nd cycle of chemotherapy, FVIII improved to >5% and hematoma reduced in size	The patient did not experience any further bleeding during the hospitalization Discharged on a prednisone taper and with an outpatient follow-up with hematology to continue rituximab cycles weekly
Kaur et al., 2018 [3]	78/F	Recurrent GI bleeding, chronic iron-deficiency anemia, T2DM, multinodular goiter	Large ecchymotic lesion along floor of mouth, ventral tongue, multiple ecchymoses over chest and upper extremities. Hemodynamically stable	1 U PRBC (due to symptomatic anemia)			Prednisone at 60 mg PO followed by rituximab	Discharged on high-dose prednisone at 60 mg PO daily with outpatient hematology follow-up Use of prednisone caused side effects, switched to rituximab two months after starting rituximab therapy, labs revealed a normal PT/PTT, FVIII level of 113%, and the Bethesda assay was not done due to the normal FVIII level No further bleeding episodes; remained asymptomatic at six months
Sheth et al., 2016 [4]	66/M	Schizophrenia, right thigh pain	Spontaneous right thigh IM hematoma	rFVIIa^a^ aPCC^a^ Surgical intervention for hematoma evacuation; profuse bleeding from incision site, PRBC and FFP AHA diagnosis made, rFVIIa was used Recurrent bleeding required aPCC to control the bleed	Suboptimal bleeding control after surgical evacuation until bypassing regimen was commenced		Prednisone 1 mg/kg/day and cyclophosphamide 2 mg/kg/day	Improved and eventually discharged to a skilled nursing facility after 35 days Readmission three weeks after discharge, readmitted for minor bleeding from surgical wound, requiring one dose of aPCC and local application of aminocaproic acid Patient discharged in stable condition after two days of inpatient stay and remained asymptomatic with regular follow-up in outpatient hematology and oncology clinic
Wool et al., 2017 [5]	73/M	Ischemic stroke, congestive heart failure, atrial fibrillation	Right thalamic hemorrhage with intraventricular extension Prolonged aPTT for eight months before presentation, but not further evaluated	4F-PCC (19.7 U/kg rFVIII (∼36 U/kg per dose) rFVIIa (30 µg/kg) TPEx	4F-PCC did not shorten aPTT rFVIII given to overcome inhibitor (total three doses) Managed with low-dose rFVIIa because of thromboembolic risk; also received dexamethasone Segmental pulmonary embolism after ~two days of rFVIIa, so switched to TPEx and received rituximab (375 mg/m^2^)		None	aPTT and FVIII values improved over time; no gross extension or growth of hematoma On 1the 6th day after admission had an episode of atrial fibrillation that progressed to cardiac arrest; FVIII increased to 275% Patient died on day 23 due to progressive shock with renal and hepatic failure
Rivera Cora et al., 2017 [6]	90/F	Multiple falls, Alzheimer’s, dementia, hypothyroidism, CKD (stage 3), lumbar compression fracture	Hematoma over right gluteal and left thigh and right shoulder	rpFVIII	Initial diagnosis of acute symptomatic anemia was treated with PRBC, FFP, and cryoprecipitate transfusions for anemia until a more definitive diagnosis After AHA diagnosis, started rpFVIII in conjunction with methylprednisolone 500 mg IV and cyclophosphamide 500 mg IV	Methylprednisolone 80 mg IV q8 initially, then methylprednisolone 500 mg IV and cyclophosphamide 500 mg IV		Controlled with rpFVIII and discharged with home care planning and subsequent hematology/oncology consultation
Singh et al., 2020 [7]	86/M	Hypertension	Oral mucosal bleeding and swelling of the oral cavity and swelling on right side of neck	aPCC at 70 U/kg Q8H Transfusion support with multiple PRBCs, FFPs, and cryoprecipitates	Prednisone 1 mg/kg daily with aPCC After extubation bleeding started again, rituximab 375 mg/m^2^ and prednisone switched to methylprednisolone 60 mg/day Intermittent oral and nasal mucosal bleeding and hematuria; given three cycles of rituximab and IV cyclophosphamide at 2 mg/kg/day and continued aPCC treatments Rituximab continued for a total of four weekly doses		Rituximab Steroids Cyclophosphamide	aPTT normalized following administration of steroids, rituximab, and cyclophosphamide Continued to improve and did not require any bypassing agents over the last few days On day 45, patient was discharged with prednisone at 60 mg PO daily with a tapering dose After four months of outpatient follow-up, laboratory investigation revealed a normal aPTT, PT, FVIII assay of 41, and FVIII inhibitor of 5.5 BU
Alidoost et al., 2020 [8]	61/M	T2DM, hypertension, hyperlipidemia, hypothyroidism, obstructive sleep apnea	Uncontrolled bleeding after fasciotomy of right leg (compartment syndrome)	PRBC, FFP, IVIg, plasmapheresis FVIII rFVIIa rpFVIII	Underwent repeat fasciotomies for compartment syndrome, unsuccessful controlling of bleeding with FVIII rFVIII with steroids unsuccessful rFVIIa with steroids administered and was unsuccessful, became blood transfusion dependent Tried rpFVIII, rituximab, IVIg, plasmapheresis Of note, had noticed increased bruising and seen previously as hematology/oncology and vascular surgery outpatient before admission and had been diagnosed with B12 and iron deficiency		Steroids (not specified)	Developed new-onset progressive thrombocytopenia Code stroke called, MRI showed innumerable peripheral enhancing mass lesions Mental status did not improve, family met with palliative care and status changed to DNR Died of pulseless electrical activity cardiac arrest Authors suggest probable underlying malignancy (colon cancer not confirmed)
Paudel et al., 2016 [9]	21/M	None	Abdominal hematoma (secondary to gunshot wounds)	rFVIIa	First presented with multiple injuries secondary to trauma, requiring extensive life-saving surgery 14 days post-op: right femoral vein and bilateral cephalic vein thrombi, treated with warfarin 24 days post-op: bleeding in GI tract, and internally, resulting in an abdominal hematoma, hemodynamic instability Warfarin stopped; multiple units of erythrocytes, FFP, and vitamin K were transfused Methylprednisolone 40 mg IV Q8H and rFVIIa stopped bleeding		Methylprednisolone 40 mg IV q 8	Once stable, rituximab infusions started and were continued once weekly for four weeks until levels normalized Bethesda assay three weeks later showed factor inhibitor of 5.2 BU, down from 12 BU
Williams et al., 2017 [10]	75/M	T2DM, CKD	Bruising and swelling of left arm, SC hemorrhaging	rFVIIa	rFVII for acute bleeding Hemolysis resolved on prednisone, but continued to be dependent on transfusions; progressive SC hemorrhaging Cyclophosphamide (100 mg/day) and rituximab therapy (375 mg/m^2 ^weekly for two weeks) were initiated		Prednisone 1 mg/kg daily Cyclophosphamide 100 mg daily Rituximab 375 mg/m^2^ for 2 weeks	Continued cyclophosphamide and prednisone at discharge Prednisone was tapered and cyclophosphamide was continued, resulting in durable remission over three years to date Patient was diagnosed with warm autoimmune hemolytic anemia and chronic lymphocytic leukemia/small lymphocytic lymphoma
Gokozan et al., 2019 [11]	76/M	Metastatic squamous cell carcinoma of lung	Extensive bruising and hematuria, deep IM hemorrhages 3 weeks before patient received nivolumab	rpFVIII 100 U/kg	aPCC (100 U/kg daily)		Steroid treatment Rituximab 375 mg/m^2 ^(4 doses weekly)	Outpatient treatment with aPCC (100 U/kg daily) until inhibitor resolved Relapse of inhibitor two weeks later eliminated with steroids and weekly rituximab 375 mg/m^2^ Complete resolution of bruising, undetectable inhibitor, normal FVIII levels four months later
Arora et al., 2016 [12]	65/M	Hypertension, COPD, renal cell carcinoma, Hodgkin lymphoma, adenocarcinoma of colon	Intrabdominal bleed (post-operative day 1, laparotomy for small bowel obstruction, despite surgical hematoma evacuation)	aPCC (75 U/kg)	aPCC		Prednisone 80 mg/day	Resolution of bleeding from surgical site; patient was discharged Remained clinically stable at 3-month follow-up with no further episodes of bleeding FVIII inhibitor disappeared and aPTT recovered to normal level (38 s)
Lewis et al., 2020 [13]	78/M	CKD, metastatic prostate cancer-Stage IV	IM hematoma of the inferior thigh and large suprapatellar hemarthrosis	PRBC infusion and 1 U of PCC rFVIIa 90 µg/kg aPCC 50 U/kg	PRBC infusion followed by 1 U PCC 12 h after PRBC transfusion, Hb inadequately increased indicating ongoing bleeding, with another unit administered without any response rFVIIa 90 µg/kg Q3H, continued for seven days (AD 1–7) Switched (due to shortage) to aPCC, 50 U/kg, Q6H (AD 7–9) when the half-life of recombinant FVIIa was completed, with taper to Q12H (AD 9–12) While on aPCC, patient was also started on rituximab (AD 8)		Rituximab ×4 weekly cycles	PTT and Hb normalized by 41 days from admission and in clinical remission for nine months with no further episodes of recurrent bleeding
Aslam et al., 2020 [14]	64/F	Pulmonary sarcoidosis, Addison disease	Left thigh hematoma	PRBC, FFP (2 U daily) FVIII (80 U/kg daily) aPCC	After mixing study treatment plan modified to include the following: aPCC, vitamin K (10 mg/day), and IV solumedrol (30 mg Q12H) three days after starting aPCC, developed large hematoma in right thigh		Methylprednisolone, 30 mg every 12 hours	Died: pulmonary embolism
Roy et al., 2020 [15]	74/F	Anemia, myasthenia gravis; history of recurrent GI bleeding for 1 month prior to diagnosis	Melena, oozing from the bowel anastomotic area (patient had presented 1 month before with acute GI bleeding, large left pectoral hematoma)	aPCC rFVIIa	One month before first presentation, patient had acute GI bleeding Endoscopy did not reveal a clear bleeding source; attributed to erosive esophagitis Patient discharged with a plan of weekly rituximab for four weeks after first admission Readmitted two days after discharge with spontaneous chest-wall hematoma, treated with aPCC and rFVIIa		Steroids Rituximab	Became unresponsive and resuscitative efforts proved to be unsuccessful Died: ICH was considered as a possible cause for her sudden death
Bennetts et al., 2018 [16]	64/F	Hypertension	Prolonged bleeding, worsening ecchymosis, and swelling of left face and neck following routine dental extraction, submandibular edema	FEIBA 125 U/kg initially, then 75 U/kg daily	Along with FEIBA, treated with 1 mg/kg prednisone, rituximab (375 mg/m^2^)		Prednisone 1 mg/kg daily Rituximab 375 mg/m^2^ (weekly, total of 4 doses)	aPTT trended downward, with inhibitor decreasing by 50% Discharged on day 10 with weekly follow-up with hematology
Taza et al., 2021 [17]	76/M	Hypertension, hyperlipidemia, COPD, papillary urothelial carcinoma	Non-traumatic ecchymosis at first presentation, then large IM hematoma in posterior right thigh	Single unit of PRBCs used on first admission then aPCC 100 mg/kg twice daily for 2 days	Admitted first time five months post-op (transurethral resection of bladder tumor) for non-traumatic ecchymosis; AHA diagnosis was made Treated with prednisone 1 mg/kg daily; aPTT improved and patient was discharged Admitted four days post discharge with severe IM hematoma in posterior right thigh Started on tranexamic acid 1300 mg three×/day and cyclophosphamide 1 mg/kg daily + 100 mg daily prednisone and aPCC 100 mg/kg twice daily for two days After two weeks, FVIII level remained persistently <1% and, as a result, cyclophosphamide was discontinued, and he was initiated on IV rituximab 375 mg/m^2^ weekly for four weeks Following initiation of rituximab, his FVIII level was noted to increase (59%) with an associated decline in his inhibitor to 4 BU and normalization of aPTT		Prednisone 1 mg/kg daily Cyclophosphamide 1 mg/kg daily Rituximab 375 mg/m^2 ^(four weeks)	Patient had underlying diagnosis of papillary urothelial carcinoma Was readmitted four days post discharge for severe IM hematoma FVIII levels took more than four weeks to rise
Singh et al., 2021 [18]	59/M	COPD, tobacco use	Skin tear evaluated in ED prior to admission; diffuse ecchymosis and petechiae throughout extremities, IM hemorrhage	No hemostatic treatment used in ED; advised to stop aspirin and discharged without symptom resolution or defined etiology aPCC (dose undefined)	High-dose prednisone (not defined) given with aPCC, then followed with steroids and rituximab until complete remission		High-dose steroids Rituximab	Complete remission after treatment with steroids and rituximab
Shen et al., 2020 [19]	74/M	Hypertension	Surgical incision sites (right upper shoulder and inguinal hernia) Hematoma in anterior soft tissues of lower mid-abdomen and moderate to large hemoperiton-eum	PRBCs and FFP Cryoprecipitate and platelets Desmopressin and rFVIIa rFVIII	Patient first diagnosed with acute blood-loss anemia secondary to surgery and treated with PRBCs and FFP Bleeding was uncontrolled and treated with PRBCs, cryoprecipitate, and platelets Hematology team consulted at this point; mixing study performed; given desmopressin (20 µg) and rFVIIa (8000 µg) Patient had similar bleeding episode earlier in year at nearby hospital (treated with PRBCs, FFP, cryoprecipitate, and aPCC); no diagnosis was made at the time Then treated with rFVIII (13,000 U/day and 8000 U at night) in addition to rFVIIa (6000 µg) and prednisone 60 mg once daily FVIII levels continued to be <1%, so rFVIII dose increased to 8000 U Q8H; rituximab 750 mg IV continued once weekly		Prednisone 60 mg orally once daily Rituximab 750 mg IV (once weekly)	Patient’s FVIII levels rose to 69% on post-op day 12; FVIII levels continued to rise and exceeded 150%, with a peak of >500% despite rFVIII dose reduction rFVIII therapy stopped and prednisone taper began On post-op day 14, patient received second dose of rituximab and continued to be stable for 1 week Patient stayed in the hospital for 21 days and was discharged to a rehab facility, where he received 1 more dose of rituximab and prednisone taper
Pinchover et al., 2020 [20]	77/M	Hypertension, mitral regurgitation post-mechanical mitral valve replacement and extramedullary C4 plasmacytoma	Soft-tissue hematoma along right posterior chest wall	5 U PRBC	rFVIIa was available, but not used Rituximab 750 mg IV weekly Daratumumab therapy for multiple myeloma continued Received PRBC throughout admission of 24 days (total of 14 U)		Dexamethasone 40 mg IV 4 days Cyclophosphamide 660 mg IV every two weeks Rituximab 750 mg IV weekly	Continued daratumumab and dexamethasone with FVIII and aPTT improvement to 33 IU/dL and 119 s, respectively, within four weeks after discharge
Hess et al., 2020 [21]	91/M	Hypertension, benign prostatic hyperplasia, atrial fibrillation, mitral valve replacement secondary to mitral stenosis	Ongoing hematuria (5 weeks) Large IM hematoma in iliopsoas muscle secondary to continued bleeding	rFVIIa 90 µg/kg Q2H for 24 h Emicizumab 3 mg/kg for 4 weeks then maintenance dose 1.5 mg/kg Q2W	To clear inhibitor treated with prednisone (70 mg) and cyclophosphamide (100 mg daily) CT revealed a large IM hematoma one week after treatment Bleeding was recurrent when off rFVIIa; started on emicizumab at this point		Prednisone 70 mg Cyclophosphamide 100 mg	Bleeding was controlled ultimately with emicizumab and remained controlled when treatment stopped
Chung et al., 2021 [22]	65/M	Hypertension, hyperlipidemia, acquired hyperthyroidism, COPD	Bilateral, upper-limb, soft-tissue hematomas requiring urgent fasciotomy on right arm for compartment syndrome Bleeding from IV sites and continuous oozing from surgical wound causing severe anemia	At presentation, PRBC, cryoprecipitate, platelet transfusions, 16 U FFP, 3 U FVIII products (unspecified) rFVIIa	Rituximab (weekly, 375 mg/m^2^ for four doses) and cyclophosphamide (daily for five days, 2 mg/kg) After inhibitor level decreased to 68 BU and FVIII activity improved to 27%, stopped rFVIIa (and PRBC) six days after stopping treatment, developed spontaneous right calf hematoma and FVIII dropped to 9% rFVIIa reinitiated at 90 µg/kg Q4H and cyclophosphamide 1.5 mg/kg daily		Steroids Dexamethasone four days then tapered to prednisone 1 mg/kg daily Rituximab 375 mg/m^2^ weekly, four doses Cyclophosphamide 2 mg/kg IV daily for 5 days	Slowly tapered off rFVIIa with no further signs of bleeding Hospital day 28 FVIII levels normalized; inhibitor was undetectable Discharged with slow taper of oral prednisone and cyclophosphamide three months post discharge, patient presented to outpatient hematology clinic and had normal FVIII level and aPTT with complete resolution of AHA
Bin Waqar et al., 2021 [23]	31/F	Sickle cell trait	Injection-site hematoma over the triceps and the site of the incision after delivery of baby via cesarean section Profuse bleeding from IV lines Hematoma anterior to the uterus’s surgical line and 2 other hematomas within the SC soft tissues with IM hematoma	FFP aPCC (50 U/kg) rFVIIa 90 µg/kg ×2 + another infusion of aPCC (50 U/kg) and 3 U RBC transfusions	Discharged home on aminocaproic acid 10 days after delivery Returned to the ED for oozing of blood from incision two days after discharge Started rituximab (375 mg/m^2^) weekly for four cycles and prednisone at (1 mg/kg) Inhibitor levels remained elevated (96 BU) and FVIII levels low (1-4%); prednisone was continued cyclophosphamide (1.5 mg/kg) added		Rituximab 375 mg/m^2^ weekly, four cycles Prednisone 1 mg/kg Cyclophosphamide 1.5 mg/kg	At 2-week follow-up, the inhibitor level decreased to 2 BU and FVIII activity increased to 76% Prednisone was later tapered, and the patient was followed up in the hematology clinic with later titers undetectable for FVIII inhibitors and normalization of PTT and FVIII
Gibson et al., 2016 [24]	32/F	Fatigue, epistaxis, bleeding when brushing or flossing teeth, rectal or vaginal bleeding, dry mouth or eyes, rashes, myalgias	Hematomas and hemarthroses on the calves, anterior thighs, and upper and lower arms, and a diffuse, confluent area of ecchymosis across the lower back	rFVIIa 90 μg/kg aPCC (75 μg/kg)	Prednisone was also given with rFVIIa; over the three days following admission, hematomas stopped expanding and hematocrit level remained stable; Discharged to complete a course of prednisone, and she continued IV injections of rFVIIa Q4H; Readmitted two days later: expanding hematoma on left thigh; aPCC given at 75 μg/kg Q6H and rituximab 375 mg/m^2^ of BSA weekly Bleeding stabilized and she was discharged		Prednisone 1mg/kg daily	Received total of six doses of rituximab and therapy with prednisone was tapered over three months eight weeks later, aPTT was 35 s and FVIII activity 54% two years after initial presentation, she has had no bleeding episodes, although aPTT remains mildly elevated at 39 seconds and FVIII level 50–60% of normal

## References

[1] Sakurai Y, Takeda T (2014). Acquired hemophilia A: a frequently overlooked autoimmune hemorrhagic disorder. J Immunol Res.

[2] Collins PW, Hirsch S, Baglin TP (2007). Acquired hemophilia A in the United Kingdom: a 2-year national surveillance study by the United Kingdom Haemophilia Centre Doctors' Organisation. Blood.

[3] Franchini M, Tagliaferri A, Mannucci PM (2007). The management of hemophilia in elderly patients. Clin Interv Aging.

[4] Coppola A, Favaloro EJ, Tufano A, Di Minno MN, Cerbone AM, Franchini M (2012). Acquired inhibitors of coagulation factors: part I-acquired hemophilia A. Semin Thromb Hemost.

[5] Collins P, Baudo F, Huth-Kühne A (2010). Consensus recommendations for the diagnosis and treatment of acquired hemophilia A. BMC Res Notes.

[6] Knoebl P, Marco P, Baudo F (2012). Demographic and clinical data in acquired hemophilia A: results from the European Acquired Haemophilia Registry (EACH2). J Thromb Haemost.

[7] Biggs R, Austen DE, Denson KW, Rizza CR, Borrett R (1972). The mode of action of antibodies which destroy factor VIII. I. antibodies which have second-order concentration graphs. Br J Haematol.

[8] Kruse-Jarres R, Kempton CL, Baudo F (2017). Acquired hemophilia A: updated review of evidence and treatment guidance. Am J Hematol.

[9] Tiede A, Collins P, Knoebl P (2020). International recommendations on the diagnosis and treatment of acquired hemophilia A. Haematologica.

[10] Janbain M, Leissinger CA, Kruse-Jarres R (2015). Acquired hemophilia A: emerging treatment options. J Blood Med.

[11] Huth-Kühne A, Baudo F, Collins P (2009). International recommendations on the diagnosis and treatment of patients with acquired hemophilia A. Haematologica.

[12] Al-Banaa K, Alhillan A, Hawa F (2019). Emicizumab use in treatment of acquired hemophilia A: a case report. Am J Case Rep.

[13] Williams C, Cable C, Choi J (2017). Treatment of chronic lymphocytic leukemia/small lymphocytic lymphoma presenting simultaneously with acquired hemophilia and warm autoimmune hemolytic anemia. Proc (Bayl Univ Med Cent).

[14] Gokozan HN, Friedman JD, Schmaier AH, Downes KA, Farah LA, Reeves HM (2019). Acquired hemophilia A after nivolumab therapy in a patient with metastatic squamous cell carcinoma of the lung successfully managed with rituximab. Clin Lung Cancer.

[15] Aslam HM, Chong T, Park J, Nicolosi T, Shah R (2020). Pulmonary embolism in acquired hemophilia A: a rare complication with factor VIII inhibitor bypassing activity therapy. Cureus.

[16] Pinchover LB, Alsharif R, Bernal T (2020). Acquired haemophilia a secondary to multiple myeloma: management of a patient with a mechanical mitral valve. BMJ Case Rep.

[17] Hess KJ, Patel P, Joshi AM, Kotkiewicz A (2020). Utilization of emicizumab in acquired factor VIII deficiency. Am J Case Rep.

[18] Bin Waqar SH, Khoury L, Hussain A, McFarlane IM (2021). Acquired hemophilia A in peripartum period: diagnostic and therapeutic dilemma. Cureus.

[19] Kaur K, Kalla A (2018). A case of acquired hemophilia A in an elderly female. J Community Hosp Intern Med Perspect.

[20] Alidoost M, Conte GA, Chaudry R, Nahum K, Marchesani D (2020). A unique presentation of spontaneous compartment syndrome due to acquired hemophilia A and associated malignancy: case report and literature review. World J Oncol.

[21] Lewis A, Joseph J, Pathak N, Baseri B, Luhrs C (2020). Acquired factor VIII deficiency in prostate adenocarcinoma presenting as multiple hematomas and hemarthrosis. SAGE Open Med Case Rep.

[22] Roy AM, Siddiqui A, Venkata A (2020). Undiagnosed acquired hemophilia A: presenting as recurrent gastrointestinal bleeding. Cureus.

[23] Chung SY, Shen JG, Sticco KL (2021). Acquired haemophilia A: successful treatment of a patient using upfront immunosuppressive therapy and haemostatic agents. BMJ Case Rep.

[24] Singh P, Gorman B, Abdelsayed N, Faris M (2021). Newly acquired factor VIII deficiency in a male ex-smoker - a case report. Ann Med Surg (Lond).

[25] Taza F, Suleman N, Paz R, Haas C (2021). Acquired Hemophilia A and urothelial carcinoma. J Community Hosp Intern Med Perspect.

[26] Bennetts NA, Mergelmeyer JE, Reimer EJ, Melville JC (2018). Initial manifestation of acquired hemophilia A after a routine tooth extraction. A case report and literature review. J Oral Maxillofac Surg.

[27] Al-Shbool G, Vakiti A (2018). Acquired hemophilia A presenting as intramuscular hematoma. J Investig Med High Impact Case Rep.

[28] Gibson CJ, Berliner N, Miller AL, Loscalzo J (2016). Clinical problem-solving. A bruising loss. N Engl J Med.

[29] Sheth C, Gill A, Sekhon S (2016). Life-threatening hemorrhage from acquired hemophilia A as a presenting manifestation of prostate cancer. J Community Hosp Intern Med Perspect.

[30] Singh N, Singh Lubana S, Dabrowski L (2020). Acquired hemophilia A: a potentially fatal bleeding disorder. Cureus.

[31] Rivera Cora NI, Irizarry Delgado F, Merle Ramírez SM, Vera Quiñones J (2017). Acquired Hemophilia A in an advanced age patient of hispanic origin: a case report. BMC Res Notes.

[32] Wool GD, Chapel D, Treml A, Miller JL (2017). Therapeutic plasma exchange as part of multimodal treatment of acquired hemophilia in a patient with concurrent acute intracerebral bleed and pulmonary embolism. Transfusion.

[33] Paudel R, Dominguez LW, Dogra P, Suman S, Badin S, Wasserman C (2016). A hematological menace: multiple venous thrombosis complicated by acquired factor VIII deficiency. Am J Case Rep.

[34] Shen M, Wang S, Sessa J, Hanna A, Axelrad A, Ali F (2020). Acquired hemophilia A: a case report. J Pharm Pract.

[35] Arora S, Goyal G, Sarmad R, Wool KJ (2016). Acquired haemophilia A: an unusual postoperative complication. BMJ Case Rep.

[36] Valentino LA, Baker JR, Butler R (2021). Integrated hemophilia patient care via a national network of care centers in the United States: a model for rare coagulation disorders. J Blood Med.

[37] Reding MT, Cooper DL (2012). Barriers to effective diagnosis and management of a bleeding patient with undiagnosed bleeding disorder across multiple specialties: results of a quantitative case-based survey. J Multidiscip Healthc.

[38] (2013). Anti-Inhibitor Coagulant Complex (FEIBA) [prescribing information]. (2013). Accessed: January 20. Highlights of Prescribing Information: Anti-Inhibitor Coagulant Complex (FEIBA).

[39] (2006). Coagulation Factor VIIa Recombinant (NovoSeven RT) [prescribing information]. (2006). Accessed: January 20. NovoSeven® Coagulation Factor VIIa (Recombinant) [Prescribing Information].

[40] (2021). Antihemophilic factor (recombinant), porcine sequence (OBIZUR) [prescribing information]. OBIZUR- Antihemophilic Factor (Recombinant), Porcine Sequence: Highlights of Prescribing Information.

[41] Ellsworth P, Chen SL, Kasthuri RS, Key NS, Mooberry MJ, Ma AD (2020). Recombinant porcine FVIII for bleed treatment in acquired hemophilia A: findings from a single-center, 18-patient cohort. Blood Adv.

[42] Kruse-Jarres R, St-Louis J, Greist A (2015). Efficacy and safety of OBI-1, an antihaemophilic factor VIII (recombinant), porcine sequence, in subjects with acquired haemophilia A. Haemophilia.

[43] Varadi K, Tangada S, Loeschberger M, Montsch P, Schrenk G, Ewenstein B, Turecek PL (2016). Pro- and anticoagulant factors facilitate thrombin generation and balance the haemostatic response to FEIBA(®) in prophylactic therapy. Haemophilia.

[44] Tarantino MD, Cuker A, Hardesty B, Roberts JC, Sholzberg M (2017). Recombinant porcine sequence factor VIII (rpFVIII) for acquired haemophilia A: practical clinical experience of its use in seven patients. Haemophilia.

[45] Pan L, Mokdad A, Turecek P, Robinson M, Jain N (2022). Assessment of interference by emicizumab in the measurement of susoctocog alfa factor VIII activity using a chromogenic assay [Abstract]. ISTH Congress Abstracts.

[46] Makris M, Iorio A, Lenting PJ (2019). Emicizumab and thrombosis: the story so far. J Thromb Haemost.

[47] Franchini M, Glingani C, De Donno G (2020). The first case of acquired hemophilia A associated with SARS-CoV-2 infection. Am J Hematol.

[48] Olsen GM, Rinder HM, Tormey CA (2021). De novo acquired hemophilia as an immune dysregulation phenomenon following SARS-CoV-2 infection. Transfusion.

[49] Hafzah H, McGuire C, Hamad A (2021). A case of acquired hemophilia A following SARS-CoV-2 infection. Cureus.

[50] Wang KY, Shah P, Roarke DT, Shakil SA (2021). Severe acquired haemophilia associated with asymptomatic SARS-CoV-2 infection. BMJ Case Rep.

[51] Helms J, Tacquard C, Severac F (2020). High risk of thrombosis in patients with severe SARS-CoV-2 infection: a multicenter prospective cohort study. Intensive Care Med.

[52] (2021). MASAC Document 257 - Guidelines for Emergency Department Management of Individuals with Hemophilia and Other Bleeding Disorders. https://www.hemophilia.org/healthcare-professionals/guidelines-on-care/masac-documents/masac-document-257-guidelines-for-emergency-department-management-of-individuals-with-hemophilia-and-other-bleeding-disorders.

[53] (2021). MASAC Document 265 - MASAC Guidelines for Pregnancy and Perinatal Management of Women with Inherited Bleeding Disorders and Carriers of Hemophilia A or B. https://www.hemophilia.org/healthcare-professionals/guidelines-on-care/masac-documents/masac-document-265-masac-guidelines-for-pregnancy-and-perinatal-management-of-women-with-inherited-bleeding-disorders-and-carriers-of-hemophilia-a-or-b.

